# Reliability and Accuracy of Peri-Interventional Stenosis Grading in Peripheral Artery Disease Using Color-Coded Quantitative Fluoroscopy: A Phantom Study Comparing a Clinical and Scientific Postprocessing Software

**DOI:** 10.1155/2018/6180138

**Published:** 2018-07-24

**Authors:** Patrick Ghibes, Sasan Partovi, Gerd Grözinger, Petros Martirosian, Fritz Schick, Konstantin Nikolaou, Dominik Ketelsen, Roland Syha, Ulrich Grosse

**Affiliations:** ^1^Diagnostic and Interventional Radiology, Eberhard-Karls-University, Tübingen, Germany; ^2^Section on Experimental Radiology, Eberhard-Karls-University, Tübingen, Germany; ^3^Department of Radiology, University Hospitals Cleveland Medical Center, Case Western Reserve University, 11100 Euclid Avenue, Cleveland, OH 44106, USA

## Abstract

**Purpose:**

To assess quantitative stenosis grading by color-coded fluoroscopy using an in vitro pulsatile flow phantom.

**Methods:**

Three different stenotic tubes (80%, 60%, and 40% diameter restriction) and a nonstenotic reference tube were compared regarding their different flow behavior by using contrast-enhanced fluoroscopy with a flat-detector system for visualisation purposes. Time-density curves (TDC), area under the curve (AUC), time-to-peak (TTP), and different ROI sizes were analyzed in three independent measurements using two different postprocessing software solutions. In addition, exemplary TDCs of a patient with a high-grade stenosis before and after stent angioplasty were acquired.

**Results:**

Color-coded fluoroscopy enabled depiction of differences in AUC and TDC between high-grade (80%), middle (60%), low-grade (40%), and nonstenotic tubes. The best correlation between high-, middle-, and low-grade stenosis was appreciated in ROIs behind the stenosis. This effect was enhanced by using longer integration times (5s, 7s) and a maximum frame rate of image acquisition for analysis (correlation coefficient rho=0.9284 at 5s). TTP showed no significant differences between high- and low-grade stenosis.

**Conclusions:**

Various clinical studies in the literature already demonstrated reproducible and reliable stenosis grading by analyzing TDCs acquired with color-coded fluoroscopy. In contrast to TTP, AUC values derived in ROIs behind the stenosis proved to be reliable parameters for stenosis grading. However, our results also demonstrate that several factors are able to significantly impact the evaluation of AUC values. More precisely, accuracy of acquired AUC values can be improved by choosing longer integration times, a large ROI size adapted to the vessel diameter, and a higher frame rate of image acquisition.

## 1. Introduction

The incidence and prevalence of peripheral artery disease (PAD) are steadily rising in developed countries [1–3]. High-grade stenosis in patients with PAD can reduce or stop the blood supply to the lower extremities, thereby causing claudication and limb ischemia. For endovascular treatment of PAD, current guidelines recommend different treatment approaches depending on the stage and location of PAD, including plain balloon angioplasty, drug-coated balloons, Nitinol-stents, drug-eluting stents, and covered stents [1, 2]. In clinical practice high-grade stenosis can be detected and quantified using different imaging modalities, including color-coded duplex sonography (CCDS), contrast-enhanced CT angiography (CTA), contrast-enhanced magnetic resonance angiography (MRA), and digital subtraction angiography (DSA) [3]. The current gold standard for the diagnosis and grading of PAD is invasive DSA which is associated with the highest sensitivity and specificity, followed by MRA and contrast-enhanced CTA. Standard DSA cannot measure the flow pattern in the target vessel due to acquisition of purely qualitative 2D image data [4]. Determination of the therapeutic improvement of the flow pattern still remains a challenge after endovascular PAD treatments [5]. CCDS would be an alternative to color-coded fluoroscopy for quantification of flow patterns. However, the accuracy strongly depends on the operator. After CCDS, further evaluation with alternative modalities is frequently required for the flow pattern in stenotic areas, thereby increasing costs [1].

Modern interventional angiographic suites offer color-coded quantitative DSA or fluoroscopy which provides real-time evaluation of pre- and posttreatment hemodynamic changes in blood vessels with high-grade stenosis using time-density curves (TDC) of angiographic images [4]. Several studies have described the benefit of color-coded DSA in the treatment and management of neurovascular diseases [6–11]. In contrast to velocity encoding in MRI or CCDS, TDC acquisition of angiographic images is technically less demanding and hence less influenced by other factors and therefore might provide another robust in vivo flow measurement [5].

To the best of our knowledge, in PAD this evolving technique has only be investigated by comparing color-coded DSA with ultrasound measurements and clinical examinations including ankle brachial index [12, 13].

The aim of this study was to compare a standard clinical postprocessing software (iFLow) with an open-source software (ImageJ) in an in vitro flow phantom, which allowed quantitative measurements in a standardized way. Therefore, reliability and accuracy of color-coded fluoroscopy derived measurements (AUC and TTP) were assessed in an in vitro flow phantom under standardized conditions.

## 2. Materials and Methods

This study was approved by the local ethics committee.

### 2.1. Stenosis Phantom

Four pieces of a flexible polyvinyl chloride hose with a length of 50 cm, an inner diameter of 10 mm, and a wall thickness of 2 mm were used. The selection of inner diameter was based on the typical iliac artery size. High-grade stenosis was defined as stenosis reducing the lumen by more than 80%. Depending on the in vivo conditions this high-grade stenosis is causing flow reduction [14, 15]. In a clinical scenario stenosis with a diameter reduction of 80% (96% cross-section) will reduce downstream blood supply significantly and hence treatment is indicated. Three hose pieces were constructed with different stenotic degrees (2 mm, 4 mm, and 6 mm inner diameter) resulting in 80% (high-grade), 60% (middle-grade), and 40% (low-grade) diameter stenosis using the thermoplastic properties of the hose material. Correct inner diameters were provided by precisely positioning round metal bars with the chosen diameters inside the hose. After heating the hose, the stenosis was formed and fixed with a cable tie to determine the inner and outer diameter. The length of the stenosis with guaranteed diameter was 4 mm (width of cable tie). A hose without stenosis served as reference. The hose pieces were fixed to a wood block and connected at both ends to the flow phantom.

### 2.2. Experimental Setup

**Figure 1:**
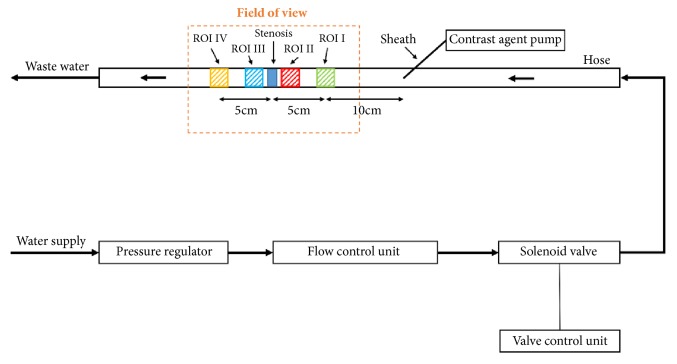
Schematic of the experimental setup.

The experimental setup consisted of a self-designed flow phantoms using contrast agent kinetics as measured with three-dimensional magnetic resonance techniques [16]. All stenotic tubes were measured with the same experimental setup, which is shown in Figure 1. The hoses were flooded with tap water and the water pressure was limited using a standard pressure regulator ranging from 4.5 bar to 1 bar. After regulating the pressure, the water was conducted via a hose to an ultrasound flow measuring unit (Sick FFU, Sick AG, Waldkirch, Germany). The flow was adjusted to 25 ml per second at an open solenoid valve, controlled by the ultrasound flow measurement unit according to physiological maximum perfusion rate in iliac arteries [17]. A measuring accuracy of less than 2% and a reproducibility of 0.5% was guaranteed based on manufacturer testing. To create pulsatile flow which reflects approximately physiological conditions a solenoid valve (R Mini-611, RPE s.r.l., Carbonate, Italy) was integrated into the setup behind the ultrasound sensor. The solenoid valve had two possible positions: completely opened or closed. These positions were controlled by a direct digital control unit [LOGO! 230RC, Siemens AG, Fürth, Germany] which allowed adjusting of opening and closing periods. The solenoid valve was directly connected with the hose and had a cycle time of 800 ms in which the solenoid valve was open for 500 ms and closed for a total of 300 ms. For contrast-enhanced fluoroscopy application of iodine-containing contrast agent was performed using a clinical standard contrast agent pump (Angiomat Illumena, Liebel-Flarsheim, Cincinnati, OH, USA). For this purpose a 6 French sheath (Terumo, Leuven, Belgium) was positioned inside the hose about 15 cm before the stenosis to ensure contrast agent distribution within the water at the time of assessment [12, 18]. In order to avoid a collection of contrast agent in the system, the contrast agent containing water behind the stenosis was collected and directed to a sewer system.

### 2.3. Imaging Protocol and Data Analysis

All measurements were performed in a state-of-the-art robotic angiography suite (Artis Zeego Q, Siemens Healthcare, Erlangen, Germany) with a flat panel detector system. The hoses with the stenosis fixed to the wood block were placed on the angiography suite table. Fluoroscopic parameters were as follows: tube voltage 56.7 kV, tube current 13 mA, and 30 frames per second over at least 10 seconds after starting the injection of undiluted contrast agent. 20 ml iodine based contrast agent (Ultravist 370, Bayer Healthcare, Leverkusen, Germany) was injected over a time period of one second. Each stenosis (80%, 60%, and 40% and no stenosis as reference) was measured independently for a total of three times. For data analysis and postprocessing of fluoroscopic images the open-source software solution ImageJ (US National Institutes of Health, Bethesda, Maryland, USA) and the commercially available software solution iFlow (Syngo, Siemens Healthcare, Erlangen, Germany) were used. Both programs enable computer calculation of color-coded maps of contrast media flow through the different stenosis models. Based on these datasets TDCs were calculated for further processing. TDCs allowed pixel-by-pixel analysis of contrast density within preselected regions of interest (ROI). Therefore, four ROIs with the same size (10x10 mm^2^) in each image series were positioned inside the hose. ROI I (reference) was located 5 cm proximal to the stenosis, ROI II was located immediately proximal to the stenosis, ROI III was located immediately distal to the stenosis, and ROI IV was placed 5 cm distal to the stenosis (Figure 1). At the hose without stenosis the ROIs were placed accordingly relative to the center of the hose.

The mean average signal (average of all pixel gray values in a ROI) in all four ROIs over the whole measurement time was calculated with ImageJ. Influences on the signal caused by X- ray absorption from the experimental setup were eliminated by setting the blank value to zero. The average signal in each ROI was integrated over different measurement times to evaluate the area under the curve (AUC). The AUC was defined as integral of the gray value over the time caused by the time-dependent contrast agent concentration in the ROI. Integration was done with QTI Plot (QTI Plot, IONDEV SRL, Bucuresti, Romania) in steps of 2 s from 1 s to 7 s (starting when the average signal in ROI I increased over 5% of the maximum possible signal by the influence of the contrast agent). For comparison purposes the alteration of the AUC over time in the ROIs II to IV relative to ROI I (relative change of gray values in percentage) was analyzed.

Time-to-peak (TTP) was analyzed as a second parameter for treatment evaluation. iFlow offers an automatic analysis of TTP, while TTP has to be manually calculated using ImageJ. The time between the start of the measurement and the arrival of the contrast agent was considered using both methods.

Additionally, the influence of the ROI size on TDC and AUC was investigated by using ROIs with varied sizes at the same position. Creation of TDC and calculating AUC was performed in the same way as calculating AUC using ImageJ.

**Figure 2:**
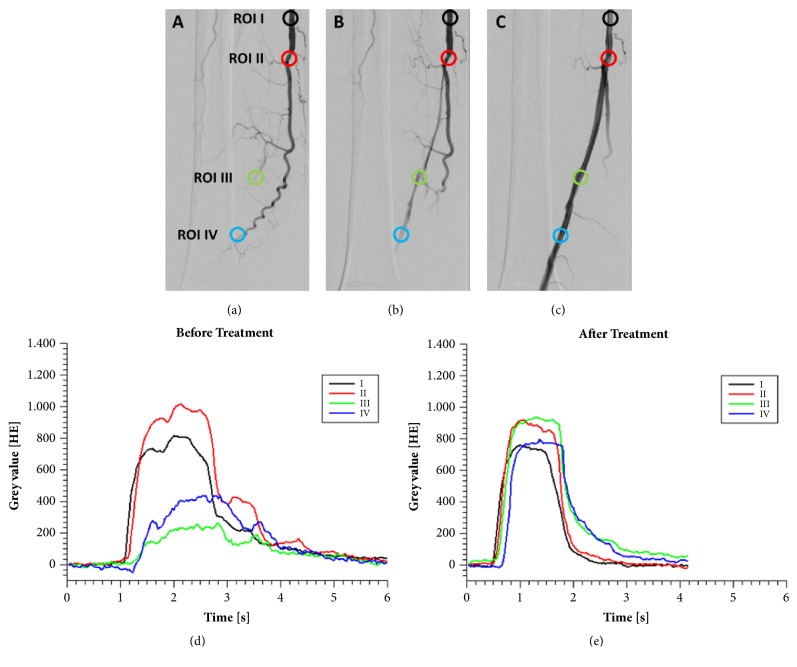
A 57-year-old patient with PAD (Rutherford 3 PAD classification) and an occlusion of the superficial femoral artery are shown (a). After balloon angioplasty a residual high-grade stenosis is visible (b) which was treated successfully by stent placement (c). Time-density curves of the treated patient case before (d) and after (e) stent angioplasty highlight the flow restriction through a high-grade stenosis.

To demonstrate the clinical applicability of the approach one patient (57 years with Rutherford 3 PAD classification) with an occlusion of the superior femoral artery (lumen reduction greater than 90%) and a residual high-grade stenosis after balloon angioplasty was investigated. Fluoroscopy was performed after balloon angioplasty with residual high-grade stenosis and after stent placement without residual stenotic disease (BioMimics 3D, Veryan Medical Ltd., Oxford, UK) (Figure 2).

### 2.4. Statistics

Statistical analysis was performed using the software package JMP (SAS, Cary, NC). The arithmetic mean of three image series and standard deviations were determined. For a priori testing the one-way analysis of variance (ANOVA) was used. The post hoc analysis was conducted using the Tukey-Kramer test. For estimation of measurement deviation, the root mean square deviation (RMSD) in gray values and its coefficient of variation in percentage were calculated for each ROI. For correlation between inner diameter of the hose and percentage of change in gray values nonparametric correlation coefficient Spearman's rho was used. Statistical significance was determined as p<0.05.

## 3. Results and Discussion

### 3.1. Results

Results of the ANOVA analysis for all different integration times, stenosis grades, and measurement ROIs using ImageJ and iFlow are listed in Tables 1 and 2, respectively. Percentage deviations of AUC in ROIs II to IV from AUC of ROI I (reference standard) measured with four different integration times using ImageJ are given in Table 1. At an integration time of 7 s the deviation of the 80% stenosis in ROI IV was −10.86% ± 6.13%. The other stenosis and the model without stenosis showed positive deviations (8.22% ± 4.69% at 60% stenosis, 23.33% ± 17.65% at 40% stenosis, and 46.75% ± 9.57% without stenosis). Statistical significant differences were detected for integration times of 3 s, 5 s, and 7 s at the ROI immediately distal to the stenotic site (ROI III) and the ROI 5 cm distal to the stenotic site (ROI IV). The post hoc test revealed significant differences for ROI III between the model without stenosis and all other stenosis models for integration times of 5 s and 7 s, respectively (p=0.0003 and p=0.03). A significant difference for the model without stenosis compared to the 80% stenosis (p=0.003) at a measurement time of 3 s was detected. The 80% stenosis model showed a significantly higher decrease in gray values compared to the other evaluated stenosis models for an integration time of 5 and 7 s (p=0.0003 and p=0.0417). At ROI IV a significant difference was seen between the model without stenosis and the 60% and 80% stenosis models for integration times of 3 s, 5 s, and 7 s, respectively (p value between 0.0008 and 0.0478). Furthermore, a significant difference between the 40% and 80% stenosis model was seen for integration times of 3 s, 5 s, and 7 s (p=0.0484, p=0.0202, and p=0.0484, respectively). No significant difference was detected between the 40% and 60% stenosis model.

In Table 2 percentage deviations of AUC in ROIs II to IV from AUC of ROI I measured with four different integration times using iFlow are listed. In ROI IV at an integration time of 7 s, the 80% stenosis showed a negative deviation of −5.67% ± 9.71%. The low-grade stenosis and the model without stenosis had positive deviations (13.00% ± 13.45% at 60% stenosis, 16.00% ± 28.51% at 40% stenosis, and 4.33% ± 4.93% without stenosis). At an integration time of 5 s the deviation at the model without stenosis was negative (−3.67% ± 5.51). 40%, 60%, and 80% stenosis degrees all revealed positive deviations (22.33%  ±  31.01% at 40% stenosis, 15.00% ± 15.13% at 60% stenosis, and 1.67% ± 11.93% at 80% stenosis). Statistically significant differences were seen for the integration times of 5 s and 7 s at the ROI immediately proximal to the stenosis (ROI II) and at the ROI immediately distal to the stenosis (ROI III). For the integration time 1 s a significant difference was seen at ROI III and at ROI IV.

**Figure 3:**
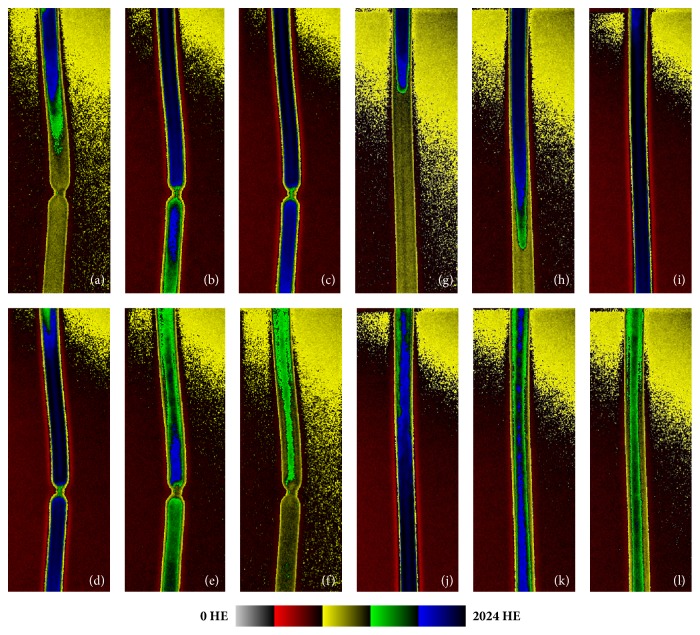
Contrast agent distribution in DSA images of the 80% stenosis (a- f) and without stenosis (g- l) converted in a color profile. Six identical moments evenly distributed over the passage of the contrast agent bolus are chosen to demonstrate the signal behavior for the 80% stenosis (a-f) and without stenosis (h-l).

**Figure 4:**
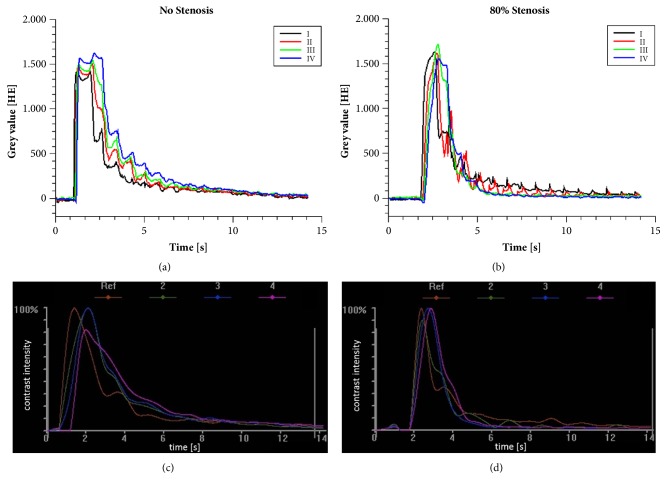
(a) Results of the flow measurements without stenosis analyzed with ImageJ. Contrast intensity curves are created for ROIs I to IV. (b) Results of the flow measurements with 80% stenosis analyzed with ImageJ. Contrast intensity curves are created for ROIs I to IV. (c) Results of the flow measurements without stenosis analyzed with iFlow. Contrast intensity curves are created for ROIs II to IV and the reference ROI (ROI I). (d) Results of the flow measurements with 80% stenosis analyzed with iFlow. Contrast intensity curves are created for ROIs II to IV and the reference ROI (ROI I). The same dataset for the 80% stenosis and without stenosis was used for flow measurements.

Figure 3 demonstrates contrast agent distribution during the periodic perfusion cycle. For this figure, 6 images per cycle were converted into a color profile using the standard Dicom viewer (Sante Dicom Viewer, Santesoft, Athens, Greece). The average signal over the time in ROIs I to IV created with QTI Plot at an 80% stenosis and without stenosis is shown in Figures 4(a) and 4(b), respectively.

### 3.2. Reproducibility of AUC Data and Correlation to Lumen Diameter

The RMSD in gray values was 154.88; 193.87; 217.02; and 230.09 for the integration times 1 s, 3 s, 5 s, and 7 s, respectively. This resulted in a coefficient of variation of the RMSD of 14.1% (1 s), 8.7% (3 s), 8.8% (5 s), and 8.9% (7 s), respectively.

A high correlation was seen between gray value changes in percentage (ImageJ) compared to inner lumen diameter at ROI II (proximal to the stenosis) for integration times of 3 s (rho=0.6478), 5 s (rho=0.7341), and 7 s (rho=0.6909). A very high correlation was seen at ROI III for the integration times of 3 s (rho=0.8421), 5 s (rho=0.8421), and 7 s (rho=0.8421) and at ROI IV for integration times of 3 s (rho=0.8421), 5 s (rho=0.9284), and 7 s (rho=0.9184). Between iFlow and ImageJ results a good correlation was appreciated for integration times of 5 s and 7 s in all evaluated ROIs with a correlation coefficient of 0.3947 and 0.5877, respectively. The results of the integration times of 1 s and 3 s showed no reasonable correlation. A high correlation was demonstrated between AUC changes in percentage (iFlow software results) compared to inner lumen diameter at ROI II (immediately proximal to the stenosis) for integration times of 5 s (rho=0.6921) and 7 s (rho=0.7692) and at ROI III (immediately distal the stenosis) for integration times of 5 s (rho=0.7163) and 7 s (rho=0.8436). No reasonable correlation was appreciated at ROI IV.

### 3.3. TTP and ROI Size Analysis

Results of the analysis of TTP using ImageJ are given in Table 3. No significant changes between low- and high-grade stenosis at ROI I to IV could be detected. TTP-values increase with an increase of the stenosis grade from ROI I to IV (0.69s to 0.84s in ROI IV). However, no significant changes between low- and high-grade stenosis could be observed (p values in ROI II and IV are 0.178 and 0.105, respectively).

Results of the analysis of TTP using iFlow are presented in Table 4. Statistically significant differences were detected for ROI II and III. Significant differences were found in post hoc analysis for ROI II and III between no stenosis and 40% stenosis and for ROI III between 40% stenosis and 80% stenosis. ROI I and IV showed no significant differences between the subgroups. No general trend from low- to high-grade stenosis was visible.

**Figure 5:**
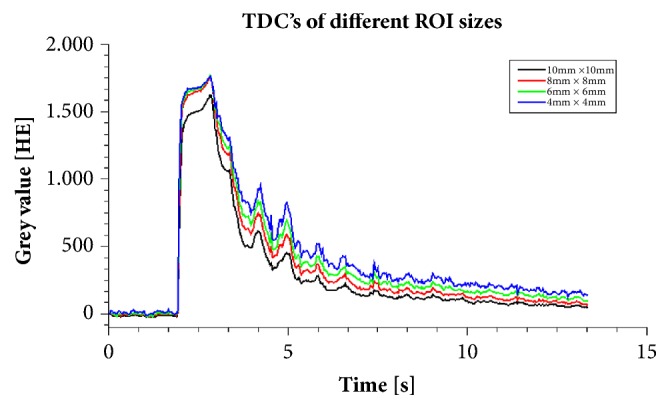
Time-density curves of four ROIs with different sizes of a nonstenotic dataset. All ROIs were positioned at the same position.

In Table 5 and in Figure 5 the influence of various ROI size's on AUC is demonstrated. A dataset without stenosis and a dataset with a high-grade stenosis was used as an example. In the nonstenotic case AUC values increase with a reduction of the area of the ROI: AUC values of a ROI with a size of 4x 4 mm^2^ increase by more than 50% in comparison to the derived AUC values with a reference ROI size (10x10 mm^2^). In the stenotic case this observed trend was much smaller, but nevertheless an increase of up to 27% could be detected.

### 3.4. Discussion

Pre- and posttherapeutic vessel stenosis grading and quantitative blood flow pattern analysis can be performed by various imaging modalities and is of utmost importance in various organ systems and diseases [19–22]. Several studies have described the ability of color-coded digital subtraction angiography to quantify or define the change in blood flow during neuroradiological interventions but the investigations of this evolving technique in PAD are currently limited. In the setting of PAD the technique has only been investigated by comparing color-coded DSA with ultrasound measurements and clinical examinations parameters, namely, ankle brachial index [6, 7]. Therefore, the aim of our study was to measure changes of contrast media kinetics in a flow phantom with a variety of precisely defined stenotic conditions using color-coded fluoroscopy. Two software solutions with varying measurement parameters were applied to conduct the analysis.

Therapeutic endovascular interventions in PAD have the goal to increase the inner luminal diameter of high-grade stenosis, thereby altering the blood flow pattern towards a more benign hemodynamic. Ultimately the endovascular intervention in PAD patient is supposed to improve blood supply to the lower extremity. For analyzing and quantifying the changes in the intraluminal contrast agent flow in this study the average signal (TDC) in a ROI was calculated. This is based on the assumption that flow alterations through a stenotic area cause changes of AUC and TTP. Gray values of a ROI provide no information about the absolute concentration of the contrast agent. However, gray values have a linear relationship to the concentration of the contrast agent. Therefore, TDCs are a surrogate marker of the contrast agent concentration within a ROI and hence a reference ROI is necessary to measure the differences of the contrast agent distribution in other ROIs located around the stenotic area. Fixed ROI sizes, which do not fill the whole cross sectional diameter can falsify results due to the great dependency of AUC values on the manual position of such ROI's in the cross-section of the vessel (see Table 5 and Figure 5) [12]. Simulations of stenotic flows have shown that the area in front and behind the stenosis is characterized by flow separation, jet flows, and recirculation flows. These phenomena can increase the mixing of water and contrast agent over the cross-section [23–25]. This explains the minor deviations of AUC measurement values at a high-grade stenosis, while the nonstenotic case presents larger deviations of AUC measurement values most likely due to the parabolic flow profile in pipes. Areas near the wall have a lower flow velocity than in the center and therefore the contrast agent is more concentrated in the center of the hose [23]. This effect is also enhanced by the 2D image mode. The exemplary dataset in Figure 5 cannot verify our assumptions but demonstrate the problem of defining the ROI size.

In this study, a difference between the flow through high-, middle-, and low-grade stenosis was appreciated using the color-coded fluoroscopy technique. The highest value of the AUC at the 80% high-grade stenosis was demonstrated in ROI I. In ROIs II to IV the deviations are lower, particularly at the integration time of 7 seconds. This likely relates to the fact that a part of the contrast agent accumulates proximal to the stenotic site thereby leading to a decreased signal distal to the stenosis. This effect persists throughout the whole measurement time and demonstrates a low standard deviation. Middle- and low-graded stenosis revealed different results since there is decreased contrast agent accumulation proximal to the stenotic site. In ROIs II to IV, the AUC values are higher than in ROI I. All AUC values with an integration time longer than 1 second are positive and the effect increases with a longer integration time. In this study an integration time of 5s to 7s revealed optimal results with significant differences between a low- and high-grade stenosis. A longer integration time does not improve the results and is associated with higher radiation exposure. Based on the assessed integration times a reliable differentiation between low-, middle-, and high-grade stenosis was feasible in this in vitro study. The best correlation between inner lumen diameter and change of gray values in percentage was seen at ROI IV at an integration time of 5s and 7s. Based on the in vitro datasets assessment of stenosis grade in PAD should be performed placing the ROI immediately distal to the stenosis (ROI III) and 5 cm distal to the stenosis (ROI IV) using integration times of 5s to 7s.

The results of the AUC analysis were verified by the TDCs as shown in the high-grade stenosis measurements (Figures 4(b) and 4(d)) when the concentration of the contrast agent in ROI I decreased more slowly compared to the ones in ROIs II to IV. The TDCs highlight the importance of the adapted integration times. High differences between TDCs of the ROIs are visible at a measurement time above 3 seconds and hence integration times shorter than 3s would degrade results.

Figure 3 shows the distribution of the contrast agent in the tubes and can help to explain the differences between high-grade and low-grade stenosis. Proximal to the stenosis no clear difference between high-grade and no stenosis is visible (Figures 3(b) and 3(h)). This is consistent with the results of finite element analysis of stenotic flow showing that the impact of the stenosis to the flow proximal to the stenotic site is minimal [26]. Distal to the stenosis two phenomena are visible. The distribution of the contrast agent in the range of ROI IV in Figures 3(c) and 3(d) is more homogenous than in Figures 3(i) and 3(j). Towards the end of the perfusion cycle the X-ray absorption of the residual contrast agent has slowed down (Figure 3(f)) compared to the reference without stenosis (Figure 3(l)). These phenomena could be explained by the strong turbulent flow fields which arise due to the collapse of the poststenotic jet flow a few centimeters distal to the stenosis. At the site distal to the stenosis admixture of water and contrast occurs [23, 24, 27]. Both phenomena lead to a reduction of the contrast agent concentration and could be an explanation for the decrease of the AUC distal to the stenosis in ROI III and IV of this in vitro study.

iFlow results reveal a reasonable differentiation between high-, middle-, and low-grade stenosis especially at ROI III (immediately distal to the stenosis) at integration times of 5s and 7s. However, results using ImageJ appear to be more convincing as they enable superior differentiation and correlation of stenosis grade and flow behavior. This was most obvious in ROI IV (5 cm distal to the stenosis) which showed the best correlation between stenosis grade and gray value changes (AUC). Figures 4(c) and 4(d) show the flow curve through an 80% stenosis and a probe without stenosis thereby revealing the reason for the difference between the analysis with iFlow and ImageJ. All measurements were performed with a frame rate of 30 images per seconds. ImageJ uses the whole frame rate for analysis. Periodic oscillations of switching the valve is clearly visible. The exact algorithm of iFlow is unknown at this point but Figure 4 indicates that the algorithm of iFlow modifies the data to smoothen out the curve (similar to a Gauss fit) which may alter the results. Fitting the curves causes also greater distances between the TDCs at the beginning of the contrast agent bolus and create artificial differences in TTP. Oscillation of valve switching during contrast agent bolus is not visible. The differences between the TDCs (high- and low-grade stenosis) are not visible and therefore the differences between the AUCs can be hardly significant.

Derived values in this study were not able to differentiate between high- and low-grade stenosis. Using ImageJ a general trend (higher stenotic grade increases TTP in ROIs distal to the stenosis) is visible, but in contrast to measurements in vivo in the literature this trend proved not to be significant [12]. Measurements with a flow phantom cannot reproduce already published results of in vivo measurements, but they show that the stenotic grade is not the only relevant factor for changing TTP. The contrast bolus peaks in TDCs verify this assumption and between low- and high-grade stenosis no shifts of the slopes were visible. Changes of TTP-values in vivo can be explained not only by a change of the stenotic grade, but also by an adjustment of hemodynamics, for example, a reduced perfusion of collateral vessels. The results of TTP-measurements using iFlow show values in the same range, but no general trend similar to the ImageJ analysis could be found. Contrary to the trend in vivo, results of the TTP-analysis using iFlow show differences in ROI II and II, but not in ROI IV. Curve fitting of iFlow also shift the peaks of TDC's and inhibit an exact measurement of TTP.

This study was performed in an experimental controlled in vitro setting and has several limitations. First, water being used as the flow medium has a different behavior at stenotic sites compared to blood. Blood has a higher viscosity and less tendencies to turbulent flow. Nevertheless under physiological conditions high-grade stenosis (80% stenosis) is known to demonstrate turbulent flows and simplification by neglecting some features of complex fluid dynamics is not uncommon in these kinds of studies [18, 19, 28, 29]. Second, the material properties of the used stenosis and the flow through the stenosis do not conform to the properties and conditions of human blood vessels. All stenotic hoses were fixed in a straight line and orthogonal to the AP-view. Blood vessels are tortuous (particularly when they are atherosclerotic), have smaller diameters, and show active movements as well as diameter changes caused by pulsatile blood flow.

The sample size (n=3) of repeated measurements for each stenotic grade was too small to make statements about reproducibility in vivo. However, due to the use of the same data for the comparative evaluation of ImageJ and iFlow, divergent results of the analysis between these software tools are most likely caused by their specific algorithms. Hence, sample size and calculated values in this study are in our opinion sufficient for a comparative analysis of these postprocessing software tools and their influence on the test parameters.

The demonstrated patient case with a treated high-grade stenosis (Figure 2) emphasizes the advantages of color-coded fluoroscopy in PAD patients. This technique can be used to assess the flow behavior in iliac and femoral arteries in “real-time” during interventional procedures. Therefore, color-coded fluoroscopy may be attractive for treatment response assessment in the angiography suite after revascularization with angioplasty and/or stenting. Nevertheless, further studies with PAD patients are necessary to evaluate the applied in vitro measurement methods under physiological conditions. The in vitro phantom used in this study simulated the iliac artery; in future studies color-coded contrast-enhanced fluoroscopy should be investigated in smaller more peripheral artery models.

In conclusion, this study demonstrates the feasibility of analyzing flow curves of color-coded contrast-enhanced fluoroscopy but also displays the influence of clinical postprocessing software tools and the setting of the test parameters on the results. Increasing reliability and accuracy of color-coded contrast-enhanced fluoroscopy imaging can be reached by using high frame-rates of fluoroscopy, a long integration time, a ROI size adapted to the cross-section diameter of the vessel, and a dedicated postprocessing software. The different flow behaviors of high-, middle-, and low-grade stenosis might provide a better treatment response monitoring after percutaneous angioplasty and/or stenting in patients with PAD.

## Figures and Tables

**Table 1:** Results of the ANOVA analysis for ROIs II to IV as analyzed with ImageJ. Percentage changes of contrast intensity curves (area under the curve) using four different integration times (1, 3, 5, and 7 seconds) are compared to a reference ROI 5 cm proximal to the stenosis (ROI I). Arithmetic mean, standard deviations, and p values (significant values are marked with an asterisk) of three repeated measurements are listed.

ROI	Integration times [seconds]	0% stenosis	40 % stenosis	60 % stenosis	80 % stenosis	p-value
II						
	1s	-2.87% (±4.85%)	0.35% (±20.6%)	-0.71% (±8.16%)	-9.99% (±6.56%)	0.71
	3s	18.67% (±3.2%)	10.81% (±14.4%)	9.68% (±4.69%)	-2,73% (±4.09)	0.1
	5s	21.12% (±5.34)	11.8% (±17.03%)	10.57% (±11.93%)	-7.87% (±2.93%)	0.06
	7s	19.88% (±5.61%)	13.59% (±19.26%)	11.17% (±13.78%)	-11.07% (±2.92%)	0.06
III						
	1s	-7.25% (±3.11%)	-12.64% (±20.74%)	-6.06% (±19.66%)	-20.83% (±6.13%)	0.62
	3s	26.06% (±3.41%)	11.73% (±10.22%)	10.73% (±4.39)	0.24% (±1.87%)	0.0048*∗*
	5s	29.75% (±4.34%)	7.84% (±11.3%)	8.49% (±1.37%)	-9.18% (±1.63%)	0.0004*∗*
	7s	28.09% (±5.65%)	5.5% (±12.78%)	7.59% (±0.97%)	-13.79% (±2.2%)	0.0007*∗*
IV						
	1s	-13.39% (±9.4%)	-16.16% (±18.65%)	-19.03% (±26.63%)	-31.68% (±11.1%)	0.62
	3s	39.38% (±5.63%)	30.32% (±15.58%)	12.51% (±9.96%)	3.52% (±6.37%)	0.0094*∗*
	5s	46.78% (±8.27%)	25.94% (±15.81%)	9.89% (±6.81%)	-5.64% (±6.1%)	0.0012*∗*
	7s	46.75% (±9.57%)	23.33% (±17.65%)	8.22% (±4.69%)	-10.86% (±6.13%)	0.0011*∗*

**Table 2:** Results of the ANOVA analysis for ROIs II to IV as assessed with iFlow. Percentage changes of contrast intensity curves (area under the curve) using four different integration times (1, 3, 5, and 7 seconds) are compared to a reference ROI 5 cm proximal to the stenosis (ROI I). Arithmetic mean, standard deviations, and p values (significant values are marked with an asterisk) of three repeated measurements are listed.

ROI	Integration times [seconds]	0% stenosis	40 % stenosis	60 % stenosis	80 % stenosis	p-value
II						
	1s	-18.67% (±17.67%)	-6% (±9.72%)	5.33% (±4.51%)	-47% (±44.19%)	0.1264
	3s	6.67% (±3.21%)	11% (±10.54%)	7.67% (±3.06%)	-10% (±18.52)	0.1608
	5s	12.67% (±2.31)	6.33% (±2.52%)	10% (±4.36%)	-13% (±12.66%)	0.0061*∗*
	7s	15% (±3%)	5% (±1.73%)	9% (±4.36%)	-17.67% (±11.68%)	0.0013*∗*
III						
	1s	-28.67% (±30.62%)	-5.33% (±6.11%)	-11% (±11.79%)	-65.67% (±39.5)	0.0474*∗*
	3s	8% (±9.64%)	19.67% (±26.39%)	12% (±4.58%)	3.33% (±17.9%)	0.5835
	5s	18.67% (±6.65%)	11% (±8.66%)	12.67% (±4.51%)	-0.67% (±12.1%)	0.02*∗∗*
	7s	23.33% (±5.77%)	5.33% (±8.39%)	10.33% (±4.04%)	-15.67% (±9.45%)	0.0012*∗∗*
IV						
	1s	-73.33% (±19.63%)	-13.67% (±18.9%)	-4% (±11.53%)	-78.33% (±31.47%)	0.0048*∗*
	3s	-23.67 % (±8.14%)	26.67% (±42.4%)	12.33% (±13.8%)	5.33% (±13.3%)	0.1422
	5s	-3.67% (±5.51%)	22.33% (±31.01%)	15% (±15.13%)	1.67% (±11.93%)	0.3526
	7s	4.33% (±4.93%)	16% (±28.51%)	13% (±13.45%)	-5.67% (±9.71%)	0.4322

**Table 3:** Results of the ANOVA analysis ROIs I to IV analyzed with ImageJ. Time-to-peak for ROI I to IV and all stenosis grades were calculated. Arithmetic mean, standard deviations, and p values (significant values are marked with an asterisk) of three repeated measurements are listed.

ROI	No stenosis [s]	40 % stenosis [s]	60 % stenosis [s]	80 % stenosis [s]	p-value
I	0.65 (±0.39)	0.39 (±0.20)	0.52 (±0.28)	0.51(±0.25)	0.745
II	0.66 (±0.37)	0.35 (±0.27)	0.56 (±0.29)	0.73 (±0.26)	0.495
III	0.66 (±0.37)	0.36 (±0.32)	0.85 (±0.26)	0.87 (±0.68)	0.178
IV	0.69 (±0.42)	0.33 (±0.25)	0.93 (±0.20)	0.84 (±0.05)	0.105

**Table 4:** Results of the ANOVA analysis ROIs I to IV assessed with iFlow. Time-to-peak for ROI I to IV and all stenosis grades were calculated. Arithmetic mean, standard deviations, and p values (significant values are marked with an asterisk) of three repeated measurements are listed.

ROI	No stenosis [s]	40 % stenosis [s]	60 % stenosis [s]	80 % stenosis [s]	p-value
I	0.95 (±0.20)	0.71 (±0.08)	0.75 (±0.08)	0.79 (±0.19)	0.308
II	1.30 (±0.14)	0.69 (±0.27)	0.92 (±0.07)	0.89 (±0.04)	0.011*∗*
III	1.30 (±0.14)	0.69 (±0.27)	1.09 (±0.12)	1.20 (±0.35)	0.010*∗*
IV	1.58 (±0.19)	0.99 (±0.55)	1.27 (±0.27)	1.29 (±0.07)	0.263

**Table 5:** Results of analyzing different ROI sizes at the same position of a nonstenotic dataset and a dataset with an 80% stenosis. Changes in AUC of ROI I to IV are compared to ROI size 10mm x 10mm and listed as AUC/REF.

Stenotic degree [%]	ROI	10mm x 10mm AUC/ REF	8mm x 8mm AUC/ REF	6mm x 6mm AUC/ REF	4mm x 4mm AUC/ REF
80	1	1.00	1.07	1.18	1.27
80	2	1.00	1.04	1.06	1.06
80	3	1.00	1.02	1.05	1.12
80	4	1.00	1.04	1.05	1.01
0	1	1.00	1.12	1.25	1.48
0	2	1.00	1.16	1.34	1.48
0	3	1.00	1.18	1.28	1.36
0	4	1.00	1.18	1.33	1.37

## Data Availability

The data used to support the findings of this study are available from the corresponding author upon request.
